# The Characteristics of Natural Killer Cells and T Cells Vary With the Natural History of Chronic Hepatitis B in Children

**DOI:** 10.3389/fped.2021.736023

**Published:** 2021-11-25

**Authors:** Yingzhi Zhou, Yi He, Yunan Chang, Xiaorong Peng, Ruiqiu Zhao, Mingli Peng, Peng Hu, Hong Ren, Min Chen, Hongmei Xu

**Affiliations:** ^1^Ministry of Education Key Laboratory of Child Development and Disorders, Department of Infection, National Clinical Research Center for Child Health and Disorders, Children's Hospital of Chongqing Medical University, Chongqing, China; ^2^Chongqing Key Laboratory of Child Infection and Immunity, Chongqing Medical University, Chongqing, China; ^3^Key Laboratory of Molecular Biology for Infectious Diseases (Ministry of Education), Department of Infectious Diseases, Institute for Viral Hepatitis, The Second Affiliated Hospital, Chongqing Medical University, Chongqing, China

**Keywords:** chronic hepatitis B, children, natural history, NK cell, T cell, immune response

## Abstract

**Background and Aims:** The immune status of children with chronic hepatitis B (CHB) in different phases is still unclear. The aim of this study was to investigate the phenotype and cytokine-producing ability of natural killer (NK) and T cells and to better understand the immune characteristics of children with different phases of CHB.

**Methods:** Treatment-naive children with CHB were divided into groups with different clinical phases of CHB. Fresh peripheral blood drawn from hepatitis B virus (HBV)-infected and healthy children was processed to perform flow cytometric analysis.

**Results:** A total of 112 treatment-naive children with CHB and 16 comparable healthy controls were included in this study. The expression of HLA-DR on NK cells and CD38 on T cells were upregulated, especially in the IA phase, in children with CHB compared with healthy controls. The ability of circulating NK cells instead of CD8+ T cells to produce IFN-γ in children with CHB was slightly increased, but TNF-α production seemed to be decreased compared with that in healthy controls. The expression of some activation markers varied among children with different phases of CHB, especially the higher CD38 expression found on T cells in the IA phase. Regression analysis revealed that IFN-γ and TNF-α production by NK cells and CD8+ T cells seemed to have positive correlations with ALT elevation and an activated status of NK cells or T cells.

**Conclusion:** NK cells and T cells tended to be phenotypically activated (especially in the IA phase) in children with CHB compared with healthy controls. However, their cytokine-producing function was not obviously elevated, especially IFN-γ production by CD8+ T cells. More studies investigating the mechanism and observing the longitudinal changes in the immune status in children with CHB are needed.

## Introduction

The clinical course of hepatitis B virus (HBV) infection may be related to the age at primary infection, transmission route, patient sex, host immune status, HBV genotype, and other factors (1). The pathogenesis of chronic hepatitis B (CHB) is complicated and remains unclear. Infants and children have a higher probability of developing chronic infection after primary HBV infection than adults (< 5%) (2, 3). There are four immunological phases in the natural history of chronic HBV infection: the immune-tolerant (IT) phase, immune-active (IA) phase, immune-control (IC) phase, and HBeAg-negative hepatitis (ENEG) phase. The four phases are mainly defined using parameters of viral replication and liver inflammation but not direct immunological biomarkers. The underlying mechanism and immune characteristics of the phases are not fully understood.

Natural killer (NK) cells are the main barrier to viral infection in natural immune responses (4). NKp30, NKp46, and HLA-DR are activated receptors expressed on the NK cell surface. Activation of NKp30 and NKp46 on NK cells was originally considered to regulate the cytotoxic and cytokine-secreting functions of NK cells (5), which are important in the recognition of, and immune response to, pathogens. The HLA-DR molecule is not only expressed on professional antigen-presenting cells as a subtype of MHC class II (6), but also is detected on T cells and NK cells as a later activation marker compared with other activation markers such as CD69 and CD25 (7). The expression of HLA-DR on NK cells can be increased in response to interaction with cytokines, infected cells, and other immune cells. HLA-DR+ NK cells are considered a functionally active subset and are increased in many infectious diseases, including HIV infection and tuberculosis (8, 9); these cells cannot only produce proinflammatory cytokines and degranulate but also may migrate through chemokine receptor CXCR3/CXCR4 and act as antigen-presenting cells, inducing the activation of T cells (10, 11). T cells play an important role in the adaptive immune response to acute HBV infection, mainly by directly killing infected liver cells or degrading cccDNA (12). CD38, as an ectonucleotidase involved in nicotinamide adenine dinucleotide (NAD)+ glycohydrolase, is a critical immune and metabolic regulatory receptor expressed on T cells (13). Through interaction with T cell receptor and regulation of NAD occupancy, CD38 mediates the early activation of T cells and affects the functional outcome of different T cell subsets (14). Epigenetic modification of CD38 could induce stable exhaustion of T cells in some chronic infection and tumor, which is refractory to PD-1-mediated functional rejuvenation (15). In HIV infection, the abnormally elevated CD38 might facilitate CD4+ T cell depletion, which might be associated with the disease progression and poor outcomes (16).

The phenotype or function of NK cells and T cells in adult CHB patients is still controversial based on data from previous studies. Peripheral NK cells and T cells have been shown to be phenotypically activated to some extent: for example, peripheral NK cells have been shown to exhibit elevated expression of the activating receptors CD69, NKp46, and NKp44, and T cells have been shown to express higher levels of the activating receptors CD38 and HLA-DR in CHB patients (17, 18). However, other studies have reported that both NK cells and T cells are functionally and partially phenotypically impaired in CHB patients: for example, both NK cells and T cells have less cytokine secretion (TNF-a and IFN-γ), and NK cells have been shown to have lower levels of activating receptors (NKp30 and NKG2D) compared with healthy individuals (19, 20). In addition, the characteristics in different phases of CHB are still unclear. Although some have reported that the function of NK cells in the IA phase were most reduced during the natural course of CHB (20), others have found increased activity of NK cells especially cytolytic activity and degranulation in the IA phase (21) and the lowest level of cytokine secretion is in the IT phase (22). Moreover, compared with CHB adults in the IT phase, children and young adults have been shown to be less functionally impaired with better cytokine secretion ability, suggesting different immune characteristics in CHB children (23). To date, however, a few studies have concentrated on elucidating the immune characteristics of children with different clinical phases of CHB. To compare the immune characteristics among the different clinical phases, we analyzed the frequencies, subsets, and phenotypes of peripheral NK cells and T cells in children with CHB.

## Methods

### Patients

Treatment-naive CHB children who met the inclusion criteria and 16 comparable healthy controls from Chongqing Medical University Affiliated Children's Hospital were included in this study. Children included in the study met the following criteria: age 1–16 years, HBsAg seropositive status over 6 months, and treatment naive at the time of enrollment. The exclusion criteria were as follows: coinfection with other hepatitis viruses (HAV, HCV, HDV, HEV), infection with human immunodeficiency virus (HIV), autoimmune or metabolic liver disease, decompensated liver cirrhosis or malignant tumor, and current immunosuppressive treatment. Informed consent was obtained from all individual participants included in the study. The definitions of the CHB clinical phases were as follows: The IT phase was characterized by HBeAg positivity with a high viral load (> 2 × 10^7^ IU/ml) and persistently normal or slightly elevated ALT < 1.5 times the upper limit of normal (ULN); the IA phase was characterized by HBeAg positivity with an elevated ALT ≥ 1.5 ULN and a viral load > 20,000 IU/ml; and the IC phase was characterized by HBeAg negativity with inactive viral replication (viral load < 2,000 IU/ml). The study was conducted according to the guidelines of the Declaration of Helsinki and was approved by the Ethics Committee of the Children's Hospital of Chongqing Medical University.

### Serological Measurements of Liver Functions, Hepatitis B Virus Markers and Hepatitis B Virus DNA

Biochemical tests were performed using routine automated analyzers. Serum HBV DNA levels were measured by real-time fluorescence quantitative polymerase chain reaction (Sansure Biotech, China), and the lowest limit of detection was 400 IU/ml. HBV markers were detected using commercial chemiluminescence microparticle immunoassay (CMIA) kits (Abbott GmbH & Co. KG, Wiesbaden, Germany). The lower limit of HBsAg detection was 0.05 IU/ml, and HBeAg positivity was assumed at values ≥ 1 S/CO (sample rate/cut off rate). The HBV genotypes were measured by real-time fluorescence quantitative polymerase chain reaction assays (Daan, China).

### Cell Surface and Cytokine Staining and Flow Cytometric Analysis

Fresh peripheral blood was drawn from HBV-infected and healthy children, and the red blood cells were lysed using NH_4_Cl lysis solution. After washing by fluorescence-activated cell sorter (FACS) buffer, leukocytes were stained with specific fluorochrome-conjugated monoclonal antibodies (mAbs) for flow cytometric analysis. For NK cell phenotypic analysis, leukocytes were stained with mAbs, including anti-CD3-PerCP5.5, anti-CD56-APC, anti-NKp30-PE, anti-NKp46-BV421, and anti-HLA-DR-FITC (BD Biosciences, USA) mAbs. For T-cell phenotypic analysis, leukocytes were stained with mAbs, including anti-CD3-PerCP5.5, anti-CD8-APC, anti-CD4-FITC, anti-CD27-PE, anti-CD45RA-BV421 (BD Pharmingen, USA), and anti-CD38-PE-Cy 7 (Biolegend, USA) mAbs. Intracellular IFN-γ-BV421 (BD Biosciences, USA) and INF-α-PE-Cy 7 (Biolegend, USA) were detected after PMA/lonomycin mixture (250×, BioLegend, USA) for 1 h and Monensin Solution (1,000×, EB eBioscience, USA) at 37°C for 4 h. Multiparameter flow cytometry was performed using a CytoFLEX flow cytometer (Beckman Coulter, USA), and data were analyzed using FlowJo software V10.

### Statistical Analysis

All statistical analyses were performed with SPSS ver. 27.0 (Chicago, IL, USA). The chi-square test was used to analyze relationships between categorical variables. Student's *t*-tests and one-way ANOVA (for normally distributed data) or the Mann–Whitney *U*-tests and Kruskal–Wallis (for non-normally distributed data) were applied when appropriate. All results were corrected for multiple comparisons using Bonferroni test. Correlations between variables were calculated by the Spearman method. Univariate and multivariate binary logistic regression analyses were performed to analyze immune variables for distinguishing the IA phase from the IT phase in children with CHB. Multivariate linear regression analysis was performed to analyze factors associated with NK cell- and T-cell-secreting cytokines. Statistical significance was defined as *p* < 0.05 (two tailed).

## Results

### Baseline Characteristics of the Study Population

A total of 112 treatment-naive CHB children (55 children in the IT phase, 45 children in the IA phase, and 12 children in the IC phase) and 16 comparable healthy controls were included in this study. The baseline characteristics of the study population are shown in Table 1. Sixty of the enrolled CHB children completed HBV genotype analysis with the result of either genotype B or genotype C. The age, sex, and HBV genotype were comparable among the CHB children of three clinical phases and healthy controls. Typical for the definition criteria, ALT levels, HBV DNA levels, and HBeAg status were different among the three CHB clinical phases (*p* < 0.05).

**Table 1:** Baseline characteristics of the study population.

**Variables**	**IT (*n* = 55)**	**IA (*n* = 45)**	**IC (*n* = 12)**	**HC (*n* = 16)**	***p***
Age (years), median (IQR)	6.89 (3.59, 9.84)	6.10 (4.05, 10.99)	5.18 (4.53, 5.18)	6.98 (4.60, 8.87)	0.953
Gender (male/female), *n*	26/29	26/19	9/3	10/6	0.305
ALT/ULN, median (IQR)	0.87 (0.69, 1.16)	3.40 (2.03, 8.32)	0.49 (0.41, 0.72)	0.40(0.38, 0.49)	**0.000**
Log_10_ HBV DNA (IU/ml), median (IQR)	8.25 (7.96, 8.46)	7.81 (6.78, 8.52)	2.60 (2.60–2.80)	NA	**0.000**
HBsAg (**+** vs. –), *n*	55/0	45/0	12/0	NA	NA
HBsAb (**+** vs. –), *n*	2/53	2/43	1/11	NA	0.649
HBeAg (**+** vs. –), *n*	55/0	45/0	2/10^*^	NA	**0.000**
HBeAb (**+** vs. –), *n*	0/55	3/42	10/2	NA	**0.000**
Genotype (B/C), *n*	10/7	24/17	2/0	NA	0.701

* *: 2 children had weakly positive HBeAg*.

*IT, immune-tolerant phase; IA, immune-active phase; IC, immune-control phase; IQR, interquartile range; ALT, alanine aminotransferase; NA, not applicable. The bold values mean p < 0.05*.

### Frequencies and Phenotypes of Natural Killer Cells and T Cells in Treatment-Naive Children With Chronic Hepatitis B

The proportions and absolute numbers of CD3–CD56+ NK cells, CD3+CD4+ T cells, CD3+CD8+ T cells, and their subsets (CD56^dim^ or CD56^bright^ NK cells, Tnaive, Tcm, Tem, or Teff T cells) were not significantly different among CHB children and healthy controls (Supplementary Figure S1).

**Figure 1:**
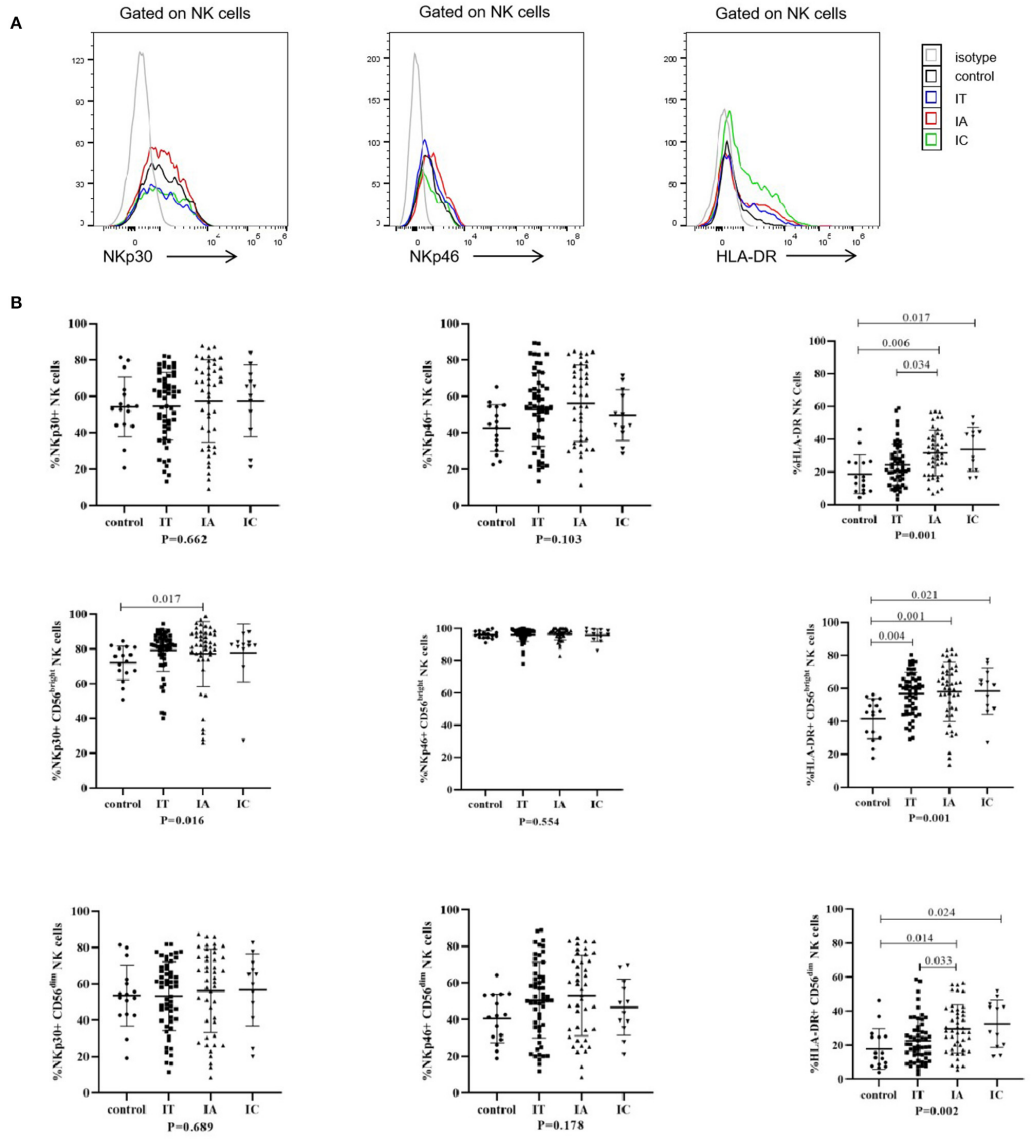
Phenotypes of natural killer (NK) cells in different clinical phases. **(A)** Representative flow histogram plots of NKp30, NKp46, and HLA-DR on NK cells. **(B)** Frequencies of NKp30, NKp46, and HLA-DR on NK cells. The *p*-values below the abscissa represent the results of the Kruskal–Wallis test of the four groups. Numbers above the lines represent the *p*-values of the multiple pairwise comparison with statistical significance. A value of *p* < 0.05 is considered statistically significant.

The phenotypes of NK cells were then analyzed (Figure 1; Supplementary Figure S2). Compared with healthy controls, HLA-DR expression on CD56^bright^ NK cells was upregulated in children with CHB in all clinical phases, and its expression on CD56^dim^ NK cells was also upregulated in children with CHB in the IA and IC phases, with statistical significance (*p* < 0.05). By comparing the different clinical phases, it was found that HLA-DR expression on CD56^dim^ NK cells and global NK cells was significantly higher in the IA phase than in the IT phase (*p* < 0.05). Similarly, compared with healthy controls, there was an increasing trend in NKp30 and NKp46 expression in children with CHB, but a statistically significant increase was only seen in NKp30+CD56bright NK cells in the IA phase (*p* < 0.05). Despite the tendency of higher expression in the IA phase, NKp30 and NKp46 expression on NK cells did not differ among the three clinical phases of CHB children (*p* > 0.05). In brief, the expression of HLA-DR, NKp30, and NKp46 in children with CHB tends to be upregulated compared with healthy controls. The expression of HLA-DR on NK cells was significantly higher in the IA phase compared with the IT phase in children with CHB.

**Figure 2:**
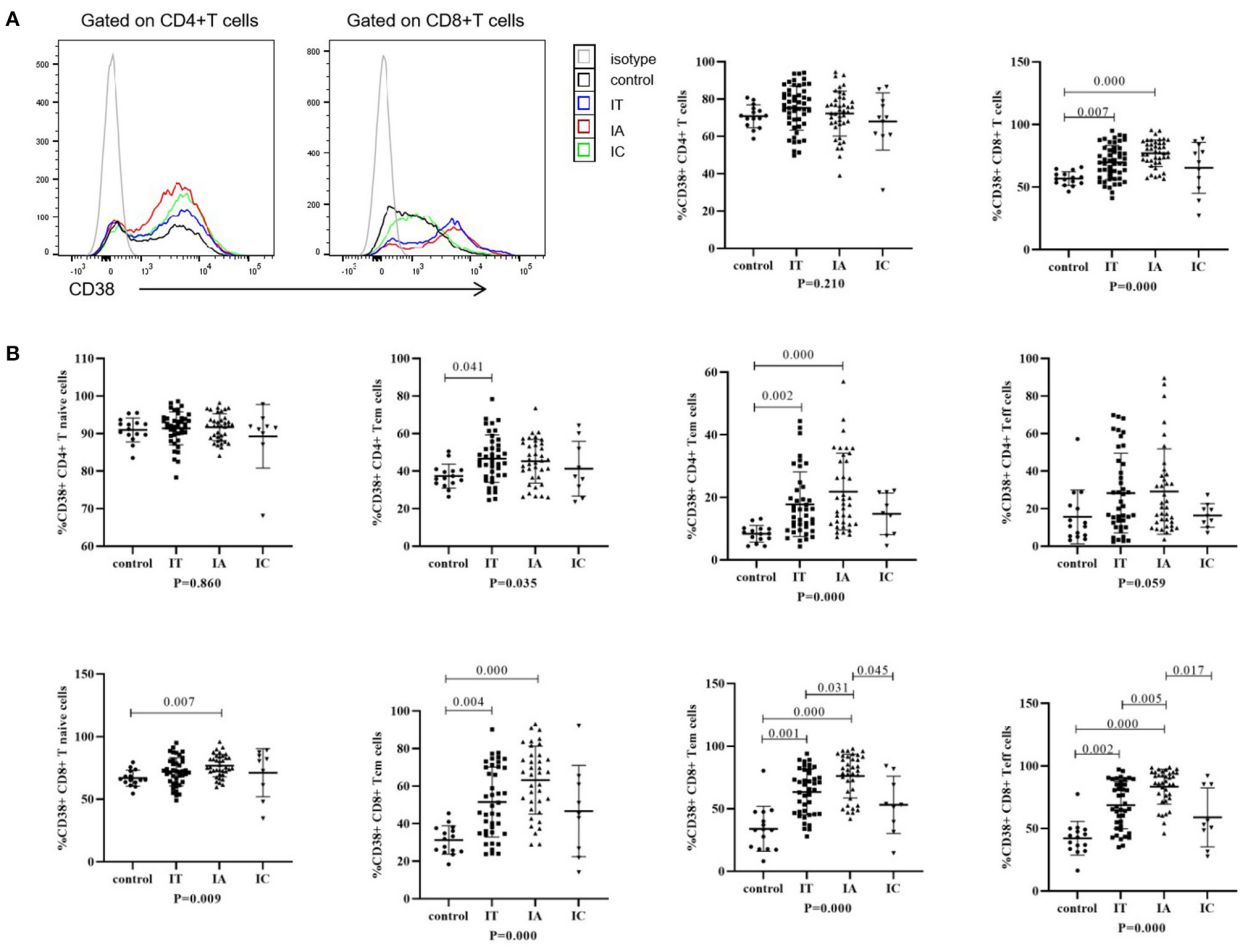
Phenotypes of T cells in different clinical phases. **(A)** Representative flow histogram plots (left) and frequencies (right) of CD38 on CD4+ T cells and CD8+ T cells. **(B)** Frequencies of CD38 on CD4+ and CD8+ memory T subsets. The p-values below the abscissa represent the results of the Kruskal–Wallis test of the four groups. Numbers above the lines represent the *p*-values of the multiple pairwise comparison with statistical significance. A value of *p* < 0.05 is considered statistically significant.

The phenotypes of CD4+ and CD8+ T cells were investigated (Figure 2; Supplementary Figure S3). Compared with the corresponding levels in healthy controls, the expression levels of CD38 on CD8+ T, CD4+ Tem, CD8+ Tnaïve, CD8+ Tcm, CD8+ Tem, and CD8+ Teff cells were significantly increased in the IA phase (*p* < 0.05); in addition, the expression levels of CD38 on CD8+ T, CD4+ Tcm, CD4+ Tem, CD8+ Tcm, CD8+ Tem, and CD8+ Teff cells were significantly increased in the IT phase (*p* < 0.05). Notably, when comparing different clinical phases, we observed that the expression of CD38 on CD8+ Tem and CD8+ Teff cells was higher in the IA phase than in either the IT or IC phase (*p* < 0.05). The expression of CD38 on global CD8+ T cells (*p* = 0.058), CD4+ Tem cells (*p* = 0.811), CD8+ Tnaïve cells (*p* = 0.239), and CD8+ Tcm cells (*p* = 0.086) tended to be higher in the IA phase than the IT phase, but the differences were not statistically significant. In general, compared with that in healthy controls, CD38 expression on T-cell subsets was upregulated in children with CHB, especially in the IA phase.

### Ability of Natural Killer Cells and T Cells Producing IFN-γ and TNF-α in Treatment-Naive Chronic Hepatitis B Children

**Figure 3:**
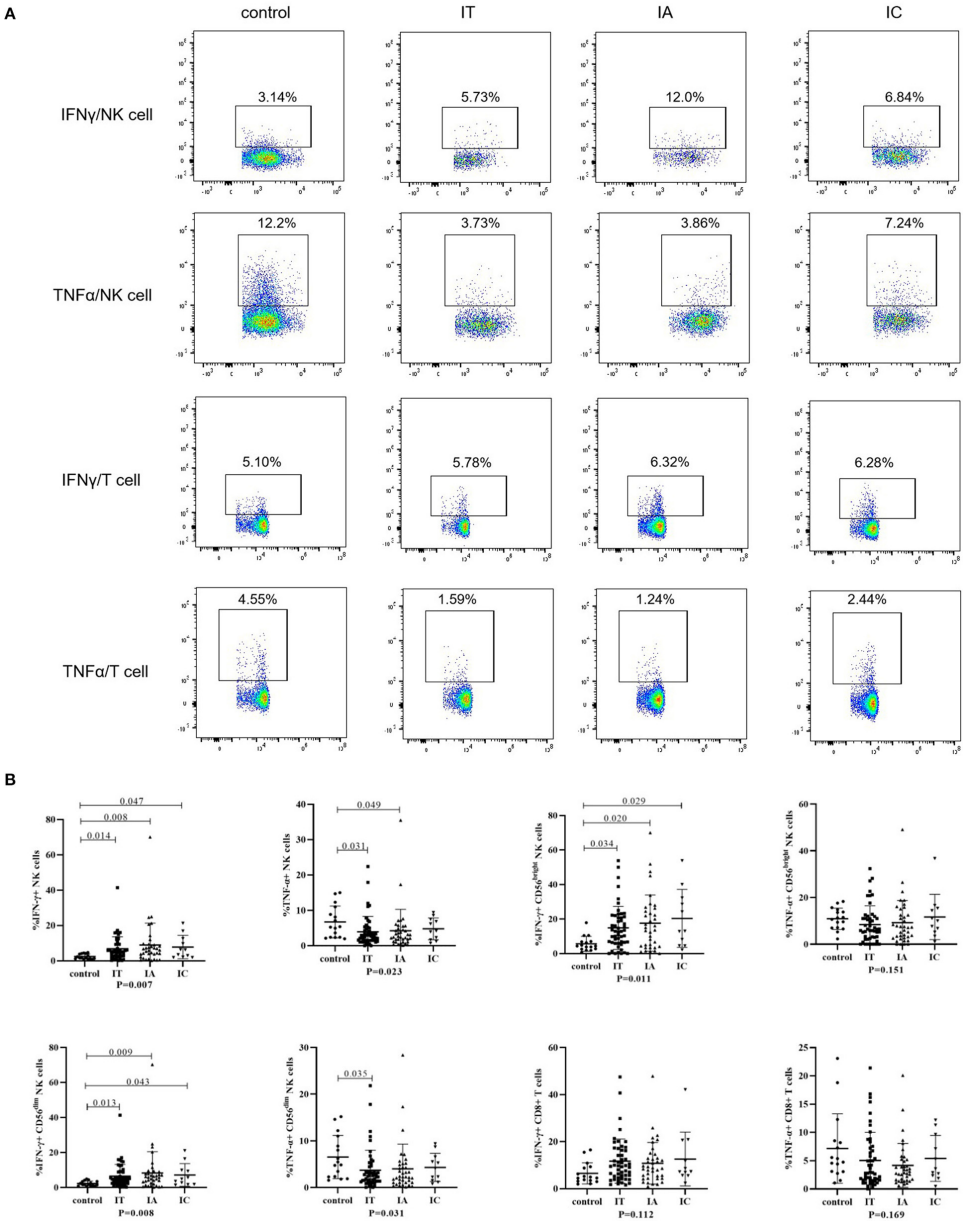
Cytokine-producing ability of NK cells and T cells in different clinical phases. **(A)** Representative flow dot plots of IFNγ and TNFα produced by NK cells and CD8+ T cells. **(B)** Frequencies of IFNγ and TNFα produced by NK cells and CD8+ T cells. The *p*-values below the abscissa represent the results of the Kruskal–Wallis test of the four groups. Numbers above the lines represent the *p*-values of the multiple pairwise comparison with statistical significance. *p* < 0.05 is considered statistically significant.

IFN-γ and TNF-α production after stimulation in children with CHB and healthy controls is shown in Figure 3; Supplementary Figure S4. IFN-γ production by global NK cells and CD56^dim^ and CD56^bright^ NK cells was increased in children with CHB, irrespective of the three clinical phases (*p* < 0.05). Conversely, TNF-α production in global NK cells and CD56^dim^ NK cells was attenuated in the IA and IT phases (*p* < 0.05) but was less impaired in the IC phase (*p* = 1.00). No significant difference in the ability of CD8+ T cells to produce IFN-γ and TNF-α was observed between different clinical phases and healthy controls (*p* > 0.05). Compared with healthy controls, an increasing trend in IFN-γ production and a decreasing trend in TNF-α production were observed in children with CHB, but the difference was not significant (*p* > 0.05).

### Evaluation of Immunological Factors for Differentiating the IA From the IT Phase in Children With CHB

**Figure 4:**
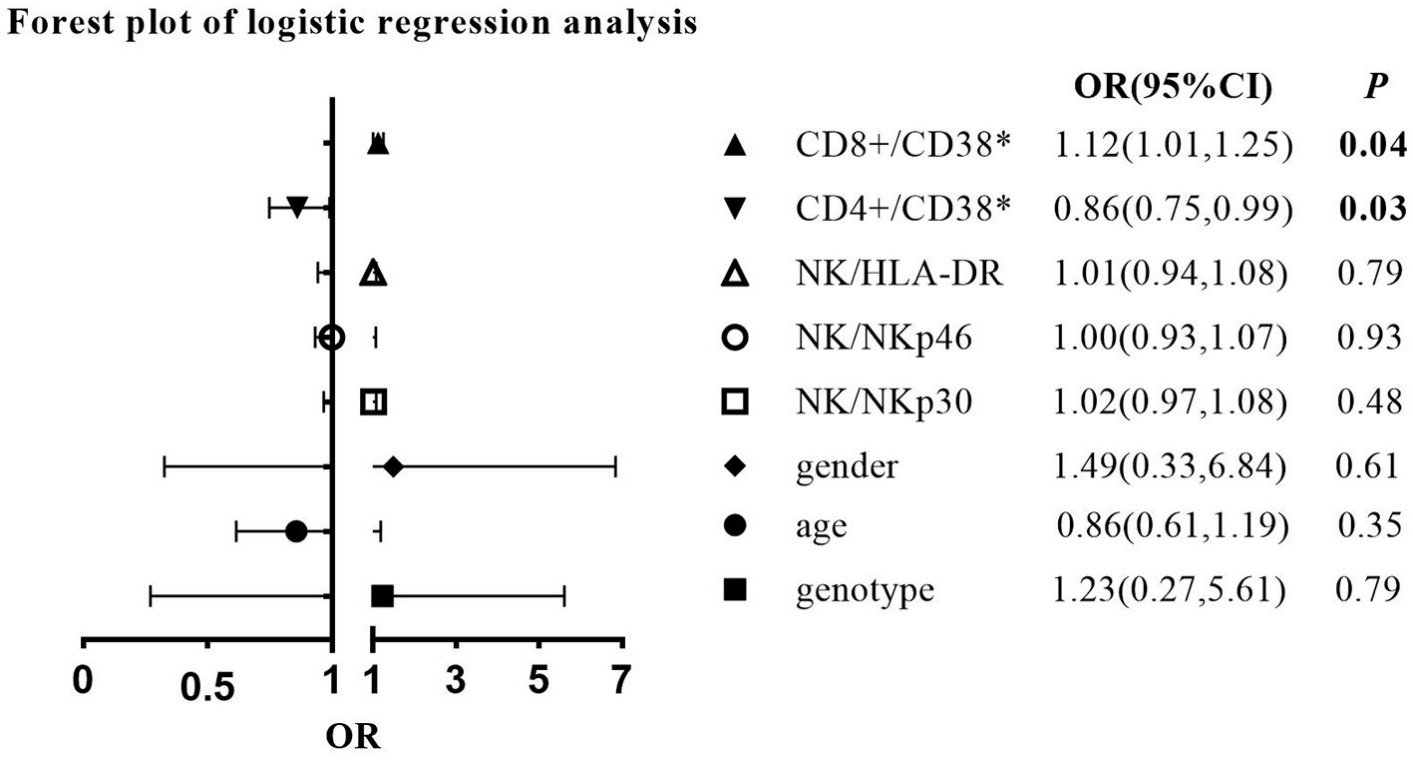
Forest plot of multivariate logistic regression analysis comparing the immune-active (IA) phase with the immune-tolerant (IT) phase (IT phase is the reference group).

To investigate independent immunological and other factors differentiating CHB clinical phases, univariate and multivariate logistic regression analyses were performed (Figure 4; Supplementary Table S1). Since the IC phase is usually not an indication for treatment, further exploration of the immune signature of the IA and IT phases might help better understand the timing and efficacy of antiviral treatment. In addition, due to the small sample size of children with IC-phase CHB in this study, we did not include the IC phase in the logistic regression analysis. Univariate analysis revealed that compared to the IT phase, the IA phase was correlated with significantly higher CD38 expression on CD8+ T cells and higher HLA-DR expression on NK cells (*p* < 0.05) (Supplementary Table S1). To adjust for other confounding factors, a multivariate logistic regression model was constructed that included age, sex, genotype of HBV, and frequencies of CD38+CD8+ T cells, CD38+CD4+ T cells, HLA-DR+ NK cells, NKp46+ NK cells, and NKp30+ NK cells (Figure 4). We excluded the ALT level, HBeAg status, and HBV DNA level because they were used in the definitions of the IA and IT phases. Among the factors, higher CD38 expression on CD8+ T cells remained an independent predictive factor for IA-phase CHB in comparison with IT-phase CHB (OR = 1.12, *p* < 0.05), while lower CD38 expression on CD4+ T cells was a predictive factor for IA phase (OR = 0.86, *p* < 0.05). The IA phase tended to be positively correlated with genotype C, female sex, a higher CD38+CD8+ T-cell frequency, HLA-DR+ NK cells, NKp46+ NK cells, and NKp30+ NK cells but negatively correlated with CD38+CD4+ T cells.

Children in the IT phase can have normal ALT levels or slightly elevated ALT levels (1). The guidelines for children with CHB reference an ALT level > 1.5 ULN as one of the considerations for treatment (24); thus, we used 1.5 ULN as the cutoff in this study to be more in line with clinical practice. However, some studies have indicated that even slightly elevated ALT levels could indicate an inflammatory and pathogenic status in children with CHB (25). To better understand the gray zone (ALT ranged from 1 to 1.5 ULN) in children with CHB, we conducted subgroup analysis comparing the strictly defined IT phase (ALT < 1 ULN) and gray zone (ALT 1–1.5 ULN). The results indicated a more activated immune status in the gray zone, with a significantly higher frequency of CD38+CD8+ T cells (Supplementary Table S2). Interestingly, we also found significantly higher CD56^bright^ NK cells but lower CD56^dim^ NK cells in the gray zone than in the strictly defined IT phase.

### Association Between Immune Cell Activity and Clinical–Virological Characteristics

**Figure 5:**
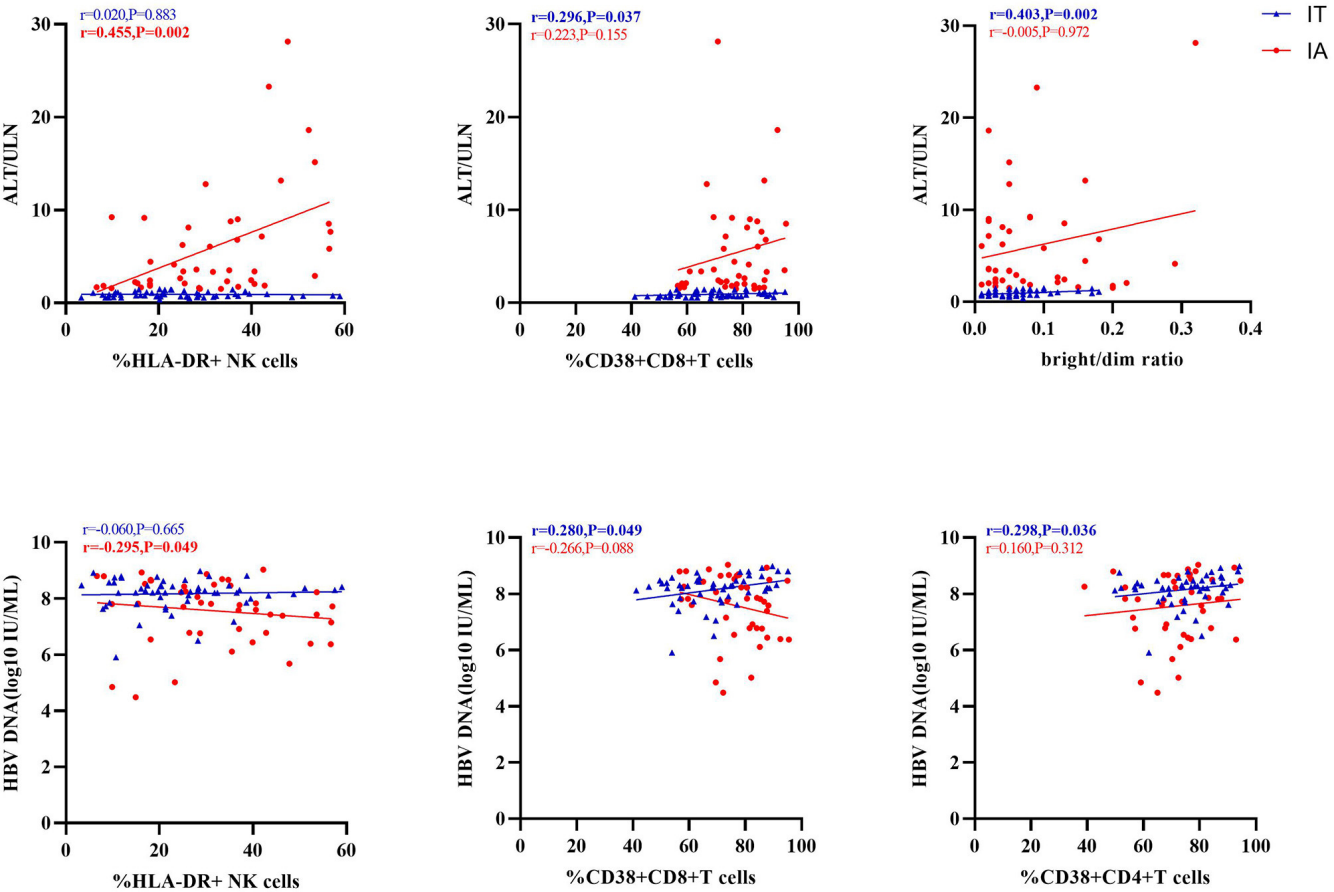
Correlation of activation markers with alanine transaminase (ALT) or hepatitis B virus (HBV) DNA levels.

We investigated the correlation of the activation receptors with serum HBV DNA and ALT in IA and IT phases of CHB patients to further explain the roles of these receptors (Figure 5; Supplementary Table S3). As shown in Figure 5, serum ALT levels had significant positive correlations with HLA-DR+ NK cells in the IA phase, as well as significant positive correlations with CD38+CD8+ T cells and bright/dim ratio in the IT phase; serum HBV DNA had significant negative correlations with HLA-DR+ NK cells in the IA phase (*p* < 0.05), as well as significant positive correlations with CD38+CD8+ T cells and CD38+CD4+ T cells in the IT phase.

**Figure 6:**
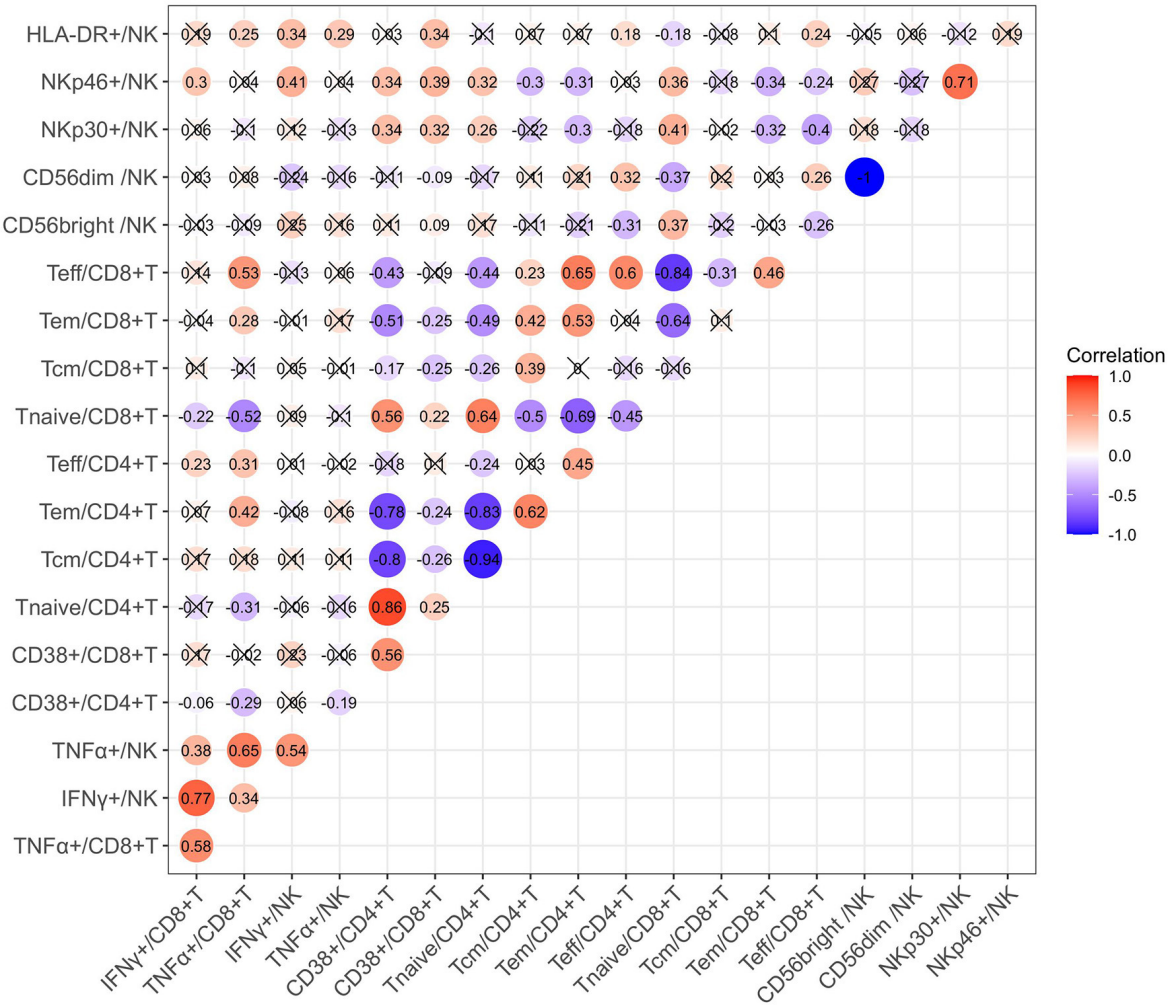
Correlation matrix of different immune cell phenotypes.

We also investigated the correlation between cytokine production and immune cell phenotypes in children with CHB using Spearman correlation analysis. As suggested by the correlation matrix (Figure 6), IFN-γ and TNF-α produced by T cells or NK cells correlated positively with each other (*p* < 0.05). When exploring the influence of cell phenotypes on cytokine production, we found the following moderated correlations: the proportion of CD8+ Teff cells correlated positively with TNF-α+ CD8 T cells, NKp46+ NK cells correlated positively with IFN-γ+ NK cells, and CD8+ Tnaive cells correlated negatively with TNF-α CD8 T cells (*p* < 0.05). In addition, correlation analysis revealed some strong correlations between cell subsets and phenotypes: the proportion of CD38+CD4+ T cells correlated positively with that of CD4+ Tnaive cells but negatively with those of CD4+ Tcm and CD4+ Tem cells (*p* < 0.05). Multivariate linear regression analysis was then used to further investigate the clinical or virological factors affecting the cytokine production by NK and T cells (Tables 2, 3). Regression analysis indicated that an increased frequency of IFN-γ+ NK cells was related to a higher ALT level and higher expression of NKp46 on NK cells but a lower frequency of CD56^dim^ NK cells (*p* < 0.05). An increased frequency of TNF-α+ NK cells was related to a higher ALT level (*p* < 0.05) (Table 2). An increased frequency of IFN-γ+ CD8+ T cells was associated with older age, higher expression of CD38 on CD8+ T cells and a lower frequency of CD4+ Tem cells (*p* < 0.05). An increased frequency of TNF-α+CD8 T cells was associated with a higher ALT level and a higher frequency of CD8+ Teff cells (*p* < 0.05) (Table 3). In summary, IFN-γ and TNF-α production by NK cells and CD8+ T cells seemed to have a positive correlation with ALT elevation and an activated status of NK cells or T cells (manifested by the higher proportions of CD56^bright^ NK cells and NKp46+ NK cells as well as CD4+ Tem, CD8+ Teff, and CD38+CD8+ T cells).

**Table 2:** Factors associated with NK cells secreting cytokines in multivariate linear regression analysis.

**Variables**		**IFN-γ+/NK**			**TNF-α+/NK**		
		**B (95% CI)**	**β**	***p***	**B (95% CI)**	**β**	***p***
Age (years)		0.40 (−0.11, 0.92)	0.16	0.125	0.26 (−0.01, 0.53)	0.19	0.058
Gender (male vs. female)		−1.23 (−4.87, 2.40)	−0.07	0.502	−0.47 (−2.45, 1.51)	−0.05	0.636
ALT/ULN		0.72 (0.30, 1.14)	0.37	**0.001**	0.31 (0.09, 0.54)	0.29	**0.007**
HBV DNA (log_10_ IU/ml)		−0.24 (−1.80, 1.33)	−0.05	0.764	−0.40 (−1.25, 0.44)	−0.15	0.346
HBeAg (**+** vs. –)		−2.23 (−12.31, 7.86)	−0.07	0.662	0.33 (−5.14, 5.80)	0.02	0.904
Genotype (genotype C is the reference group)	B	−1.71 (−6.84, 3.43)	−0.08	0.511	−2.29 (−5.02, 0.44)	−0.22	0.099
	N	0.11 (−4.81, 5.03)	0.01	0.964	−0.70 (−3.42, 2.03)	−0.07	0.612
NK/lymphocyte (%)		0.27 (−0.10, 0.63)	0.19	0.153	0.11 (−0.09, 0.31)	0.14	0.287
CD56^dim^/NK (%)		−0.47 (−0.91, −0.04)	−0.26	**0.034**	−0.19 (−0.43, 0.05)	−0.19	0.121
NKp30+/NK (%)		−0.09 (−0.22, 0.04)	−0.19	0.173	−0.04 (−0.10, 0.03)	−0.15	0.298
NKp46+/NK (%)		0.22 (0.09, 0.36)	0.47	**0.002**	0.05 (−0.02, 0.13)	0.21	0.163
HLA-DR+/NK (%)		0.01 (−0.12, 0.14)	0.01	0.886	0.05 (−0.02, 0.12)	0.14	0.182

*NK^bright^ (%) were excluded from the regression model due to multicolinearity*.

*N, not detected; CI, confidence interval; ALT, alanine aminotransferase. The bold values mean p < 0.05*.

**Table 3:** Factors associated with T-cell-secreting cytokines in multivariate linear regression analysis.

**Variables**		**IFN-γ+/CD8+T**			**TNF-α+/CD8+T**		
		**B (95% CI)**	**β**	***p***	**B (95% CI)**	**β**	***p***
Age (years)		1.99 (0.68, 3.31)	0.73	**0.004**	0.54 (−0.08, 1.16)	0.42	0.088
Gender (male vs. female)		2.62 (−2.40, 7.63)	0.13	0.301	0.14 (−2.22, 2.51)	0.02	0.904
ALT/ULN		2.62 (−2.40, 7.63)	0.24	0.071	0.28 (0.01, 0.55)	0.27	**0.045**
HBV DNA (log_10_ IU/ml)		0.53 (−0.05, 1.10)	0.35	0.099	0.31 (−0.75, 1.37)	0.12	0.561
HBeAg (**+** vs. –)		1.88 (−0.36, 4.12)	−0.26	0.202	−1.01 (−7.38, 5.36)	−0.06	0.752
Genotype (genotype C is the reference group)	B	−8.70 (−22.2, 4.81)	−0.30	0.058	−1.42 (−4.53, 1.70)	−0.14	0.367
	N	−6.40 (−13.01, 0.21)	0.11	0.537	1.47 (−1.79, 4.73)	0.16	0.370
CD8+T/lymphocyte (%)		2.14 (−4.77, 9.05)	−0.02	0.886	−0.03 (−0.21, 0.14)	−0.05	0.717
Tcm/CD8+T (%)		0.30 (−0.18, 0.77)	0.20	0.218	0.06 (−0.16, 0.29)	0.09	0.585
Tem/CD8+T (%)		−0.17 (−0.86, 0.51)	−0.07	0.612	0.27 (−0.05, 0.59)	0.22	0.099
Teff/CD8+T (%)		0.18 (−0.08, 0.43)	0.24	0.164	0.14 (0.02, 0.26)	0.40	**0.026**
CD38+/CD8+T (%)		0.41 (0.05, 0.77)	0.55	**0.025**	0.05 (−0.12, 0.22)	0.15	0.539
CD4+T/lymphocyte (%)		−0.37 (−0.85, 0.11)	−0.25	0.128	−0.02 (−0.24, 0.21)	−0.03	0.878
Tcm/CD4+T (%)		−0.33 (−1.01, 0.36)	−0.28	0.343	−0.21 (−0.54, 0.11)	−0.38	0.190
Tem/CD4+T (%)		−1.56 (−2.75, −0.37)	−0.66	**0.011**	−0.31 (−0.87, 0.26)	−0.28	0.280
Teff/CD4+T (%)		0.44 (−2.35, 3.23)	0.04	0.753	0.00 (−1.31, 1.32)	0.00	0.997
CD38+/CD4+T (%)		−0.25 (−1.02, 0.52)	−0.28	0.515	−0.11 (−0.48, 0.25)	−0.27	0.536

*CD8+Tnaive and CD4+Tnaive cells were excluded from the regression model due to multicolinearity*.

*N, not detected; CI, confidence interval; ALT, alanine aminotransferase; HBeAg, HBV e antigen. The bold values mean p < 0.05*.

## Discussion

The change in immune status in CHB is dynamic and continuous during the disease course (26). The current definitions of the clinical phases might not always be accurate and unanimous in predicting the immune status. Our study explored and summarized some of the distinct immune characteristics of CHB children with different clinical phases of CHB.

First, the frequencies and absolute numbers of NK cells and T cells or their subsets in HBV-infected children might not obviously differ among different clinical phases (Supplementary Figure S1), in agreement with one previous study in adults (19). Therefore, direct measurement of the frequencies and numbers of peripheral NK cells and T cells might not be sufficient to distinguish different disease phases in pediatric CHB patients.

Second, in children with CHB in both the IA phase and the IT phase, the expression of HLA-DR on NK cells (Figure 1) and the expression of CD38 on T cells (Figure 2) were higher than those in healthy controls. The activated NK cell and T-cell phenotypes in children with CHB were not significantly impaired or even increased in children with CHB. Moreover, the activation of NK cells and T cells was different across the clinical phases of CHB and more obvious in the IA phase than in the IT phase. The difference was further examined by multivariate logistic regression analysis comparing the IA phase and the IT phase, which identified a higher frequency of CD38+CD8+ T cells as the independent predictive factor for the IA phase (Figure 4). Moreover, in the subgroup analysis of the IT phase, the frequency of CD38+CD8+ T cells was still significantly higher in the gray zone (ALT within 1–1.5 ULN) compared with the strictly defined IT phase (ALT < 1 ULN), suggesting CD38 as the early marker of the transition from the IT to the IA phase. It is still unclear whether children with CHB in the gray zone would derive more benefit from antiviral therapy than conservative observations indicate. However, our study indicated that the immunological characteristics of the gray zone and strictly defined IT phases were different to some extent (Supplementary Table S2), a phenomenon that should be recognized in reconsidering the indication for antiviral therapy. Currently, only limited data are available to summarize the characteristics of HLA-DR+ NK cells in CHB. Studies have reported the association of HLA-DR+ NK cells with increased IFN-γ production in infectious and autoimmune diseases (27–29). We also found a positive relation between the frequencies of HLA-DR+ NK cells and IFN-γ+ NK cells in children with CHB, but the correlation coefficient was low (*r* = 0.34). The increased expression of HLA-DR without a significant increase in IFN-γ production in children with CHB might indicate that the activation of NK cells might be insufficient for viral clearance during chronic viral infection. CD38, an important immune and metabolic modulator of T cells, has been reported to have complex roles in T-cell activation and different functional outcomes (30). In chronic HBV infection, Cao et al. found that CD38+CD8+ T cells were increased in CHB patients compared with healthy controls (31). In agreement with these findings, we also found increased expression of CD38 on T cells in children with CHB compared with healthy controls. However, it remains unclear whether the increase in CD38+ T cell subsets is a favorable prognostic factor in CHB. The expression of CD38 on CD4+ and CD8+ T cells might have different roles in CHB. We found that CD38+CD8+ T cells were positively correlated with IFN-γ production by CD8+ T cells (Table 3). However, we also found that CD38+CD4+ T cells had a negative correlation with cytokine production (Figure 6; Table 3) and a positive correlation with the HBV DNA level (Figure 5). Activated CD38+CD4+ T cell subsets without cytokine production might contribute to HBV persistence. The roles of HLA-DR+ NK cells and CD38+ T cells in CHB patients need to be further explored.

Finally, we found that the ability of circulating NK cells to produce IFN-γ was increased, but their ability to produce TNF-α was decreased in children with CHB compared with healthy controls. A similar trend was observed for T cells, but the trend did not achieve statistical significance (Figure 3). Our findings suggested that cytokine production was not totally suppressed in children with CHB. NK cell and T-cell cytokine production (both IFN-γ and TNF-α) in CHB adults was reported to be decreased compared with that in healthy controls (20, 22). The reason for this discrepancy is unknown but could be partially due to age, stimulation method, or other confounding factors. Moreover, a possible explanation may be that pediatric patients have a less compromised or exhausted HBV-specific immune response than adult patients (23). To identify the independent factors related to cytokine production, further multivariate linear regression analysis was performed in this study (Tables 2, 3), showing that both T and NK cells producing IFN-γ and TNF-α seemed to be positively correlated with an activated status of immune cells (such as NKp46+ NK cells and CD38+CD8+ T cells). We also found that TNF-α production was positively associated with the ALT level but did not find a significant relationship between IFN-γ-producing CD8+ T cells -and the ALT level, which possibly suggested a more prominent role of TNF-α in liver inflammation. One previous study also indicated that TNF-α production by T cells was more strongly related to liver injury without viral clearance (32). The regulatory mechanism and the balance of IFN-γ and TNF-α in CHB patients need to be further elucidated.

One limitation of our study was that we could not detect the HBV-specific immune response due to the limited amount of blood samples we could obtain from children. Multispecific and strong T-cell responses were increased and crucial in self-limiting acute HBV infection, while the frequency of HBV-specific T cells was low in chronic infection, which suggested the importance of exploring the HBV-specific immune response (33). A positive correlation between the activation of NK cells or T cells and the HBV-specific immune response was reported in adult patients (34), but the correlation remains unclear in children with CHB. Second, we could not collect liver samples to verify our findings in peripheral blood and explore the local immune status in the liver. Previous studies have indicated that the status of liver-derived lymphocytes tends to be more activated and related to the level of HBV replication than that of peripheral lymphocytes (21, 35). However, peripheral blood collection is still more feasible and applicable than liver biopsy in the clinic. Third, adaptive-like expansions of NK cell subsets are known to occur in response to human cytomegalovirus (CMV) infection (36, 37). For some children in our study, clinicians excluded CMV infection based on their age characteristics and clinical manifestations, so we could not obtain exact laboratory evidence. The added effects of CMV on the formation and maturation of NK cell receptors in children with CHB should be further evaluated.

Our study is one of the first studies investigating the immune characteristics of different clinical phases in children with CHB, which supplements the lacking research data of children in this field. Besides, the findings may be important in exploring the mechanism of HBV infection chronicity and the optimal indication to start treatment in CHB management. In summary, the study found that NK cells and T cells tended to be phenotypically activated (especially in the IA phase) in children with CHB compared with healthy controls. Previous studies have reported a relatively suppressive immune status in adults with CHB compared with healthy controls (19, 20), while our results indicated that the status seemed different in children with CHB, suggesting that the HBV-specific immune response in children with CHB was probably less exhausted. Moreover, the levels of some activation markers, such as HLA-DR on NK cells and CD38 on T cells, varied among children with different stages of CHB. However, no single cytokine or cell phenotype was sufficient to summarize the immune signature and separate the natural history of HBV infection because it was better to understand the process as an interaction network with a variety of immune effectors (38). In addition, it was still unclear whether the activation markers were favorable prognostic factors. Even though NK cells and T cells were significantly phenotypically activated, their cytokine-producing function was not obviously elevated, especially IFN-γ production by CD8+ T cells. More studies investigating the mechanism and observing the longitudinal changes in the immune status in children with CHB are needed.

## Data Availability Statement

The raw data supporting the conclusions of this article will be made available by the authors, without undue reservation.

## Ethics Statement

The studies involving human participants were reviewed and approved by Ethics Committee of the Children's Hospital of Chongqing Medical University. Written informed consent to participate in this study was provided by the participants' legal guardian/next of kin.

## Author Contributions

HX and MC designed the study. YZ and YH performed the experiments and wrote the manuscript. YH, YC, XP, and RZ analyzed the data and revised the manuscript. HX, HR, MC, MP, and PH revised the manuscript and edited the language. All authors have read and approved the final manuscript.

## Funding

This study was funded by the National Science and Technology Major Project of China (2017ZX10202203007008).

## Conflict of Interest

The authors declare that the research was conducted in the absence of any commercial or financial relationships that could be construed as a potential conflict of interest.

## Publisher's Note

All claims expressed in this article are solely those of the authors and do not necessarily represent those of their affiliated organizations, or those of the publisher, the editors and the reviewers. Any product that may be evaluated in this article, or claim that may be made by its manufacturer, is not guaranteed or endorsed by the publisher.

## Supplementary Material

Supplementary Figure S1 Frequencies and absolute number of NK cells, CD4+ and CD8+ T cells in different clinical phases.

Supplementary Figure S2 Representative flow plots of the gating strategy used for phenotype assessment of NK cells.

Supplementary Figure S3 Representative flow plots of the gating strategy used for phenotype assessment of T cells.

Supplementary Figure S4 Representative flow plots of the gating strategy used for functionality assessment of NK cells and CD8+ T cells.

Supplementary Table S1 Univariate and multivariate analysis of variables associated with clinical phases (IA phase vs. IT phase) in CHB children.

Supplementary Table S2 Comparison of immune characteristics between gray zone and strict IT phase.

Supplementary Table S3 Correlation analysis of the activating NK and T cell subsets with serum HBV DNA and ALT levels.
