# Supplementary figures and images for: The Characteristics of Natural Killer Cells and T Cells Vary With the Natural History of Chronic Hepatitis B in Children
Source: Front Pediatr. 2021 Nov 25;9:736023. doi: 10.3389/fped.2021.736023 (PMC8656424; doi:10.3389/fped.2021.736023)

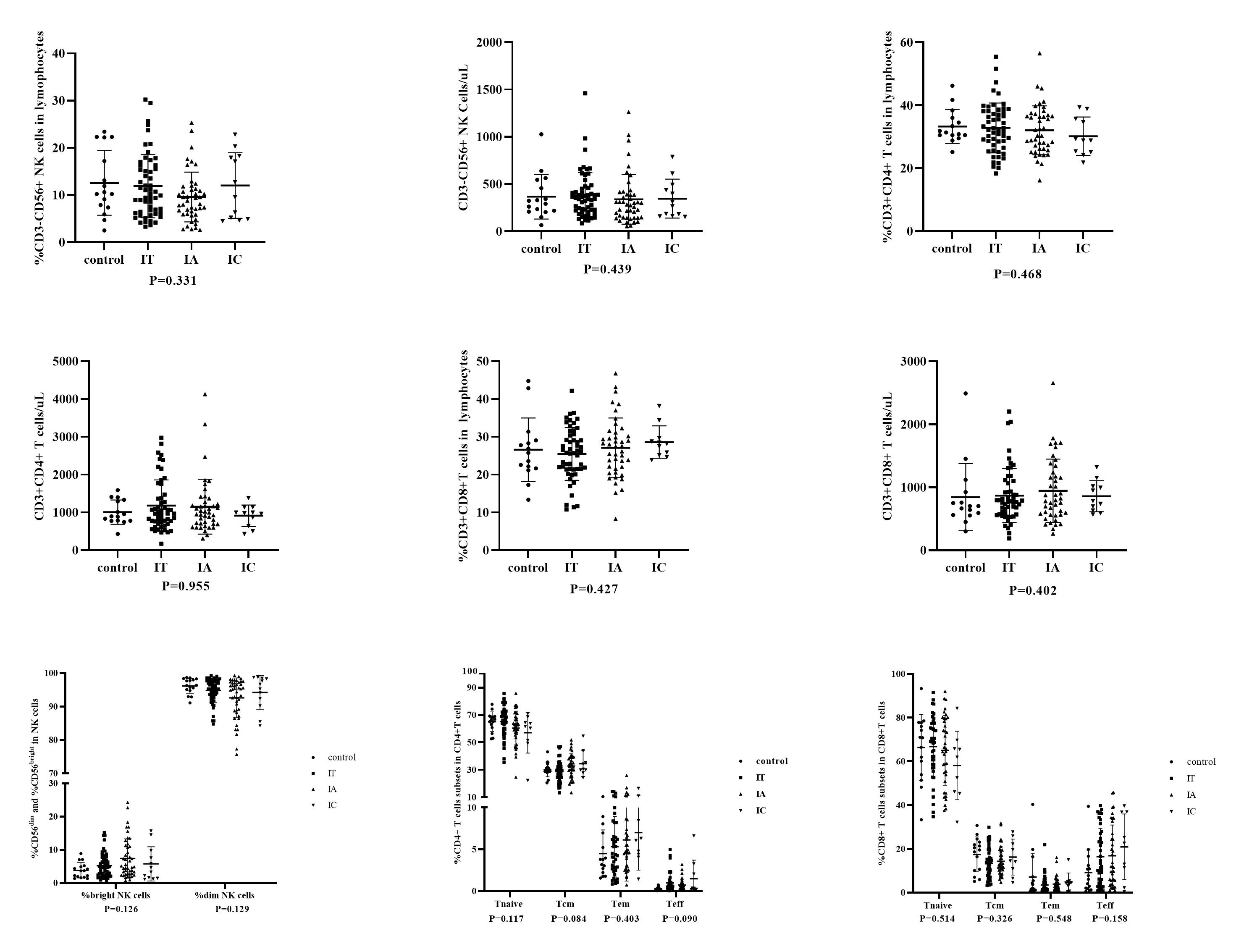

Supplement: Supplementary Figure S1 — Frequencies and absolute number of NK cells, CD4+ and CD8+ T cells in different clinical phases. [file Image_1.JPEG]

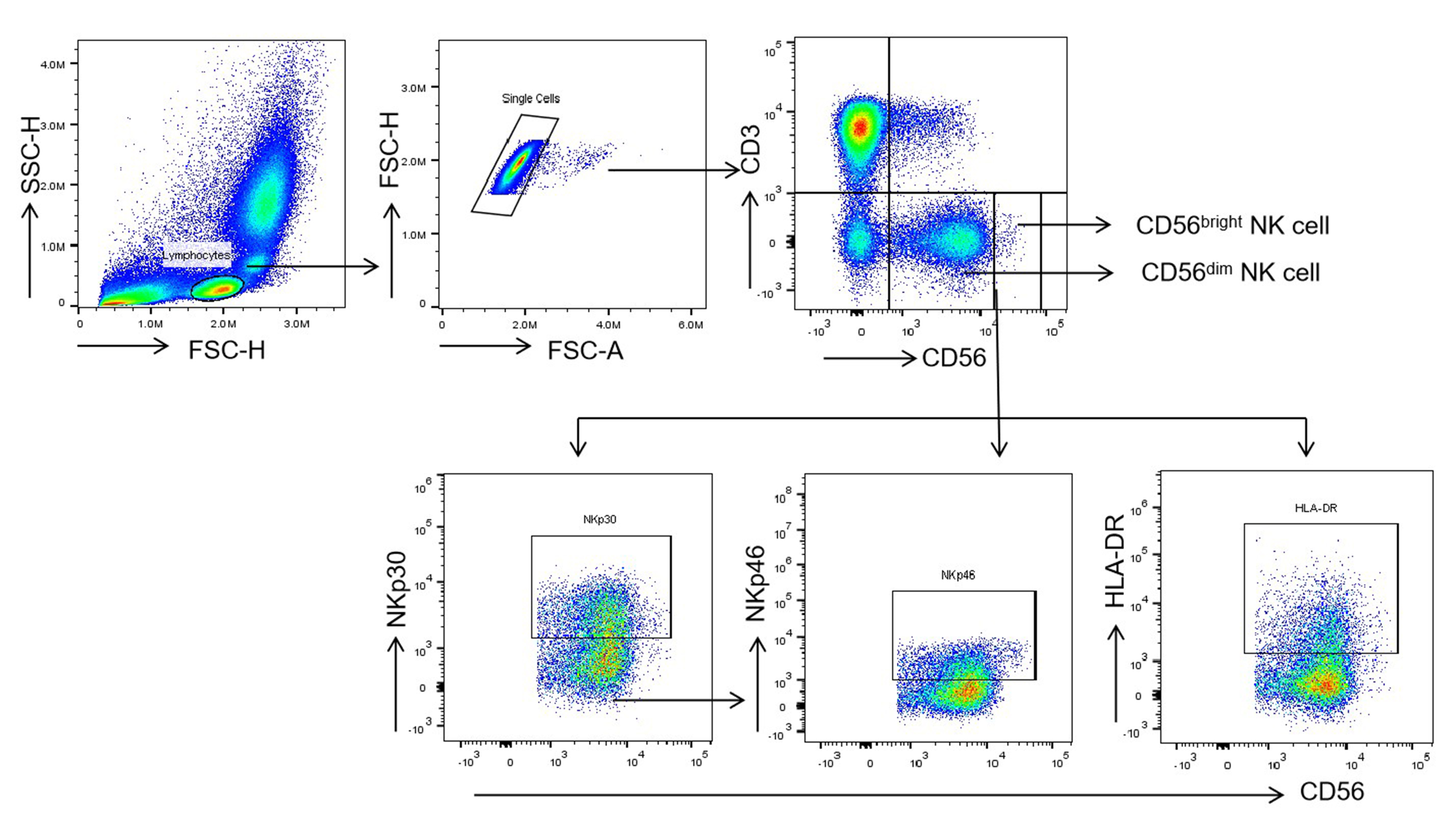

Supplement: Supplementary Figure S2 — Representative flow plots of the gating strategy used for phenotype assessment of NK cells. [file Image_2.JPEG]

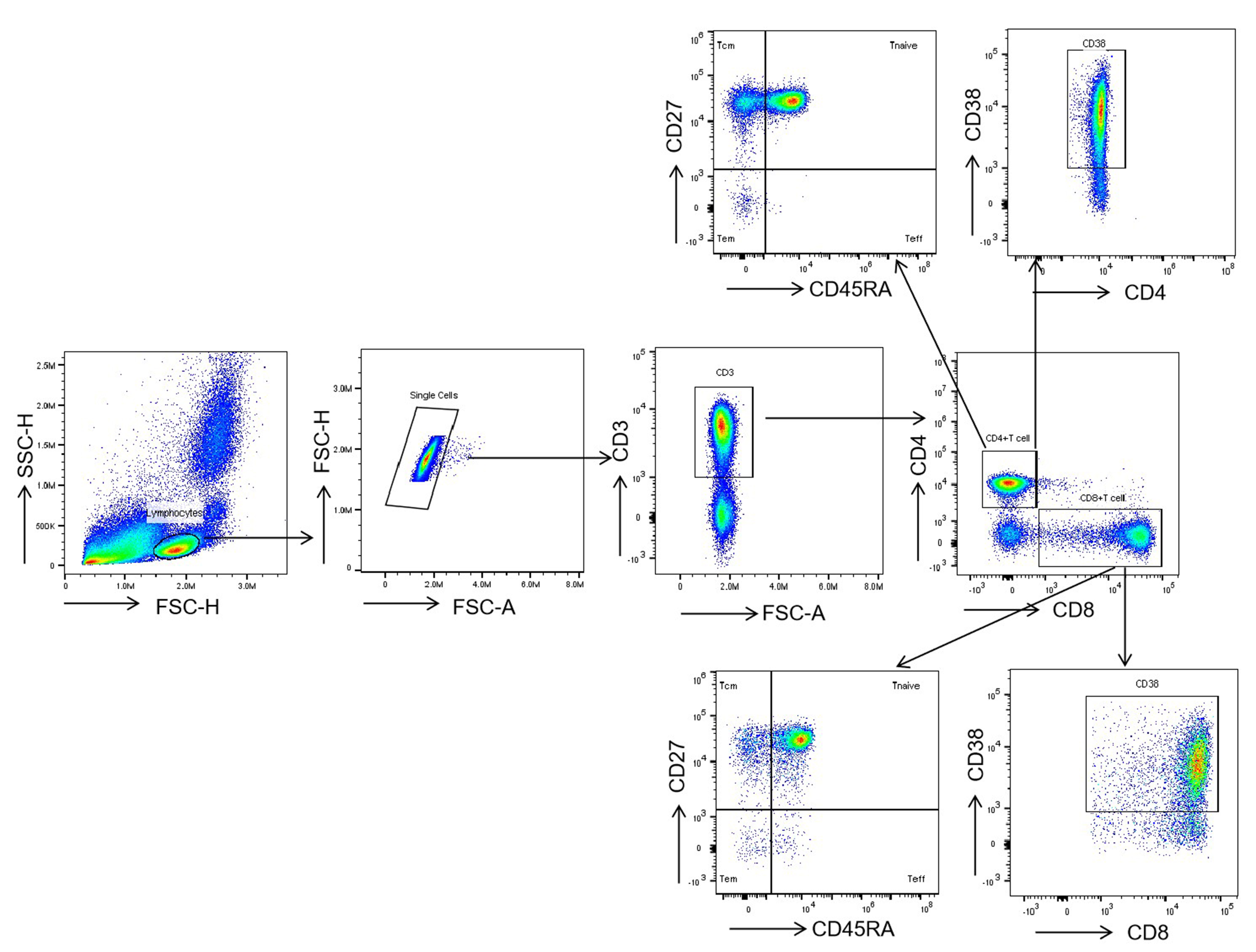

Supplement: Supplementary Figure S3 — Representative flow plots of the gating strategy used for phenotype assessment of T cells. [file Image_3.JPEG]

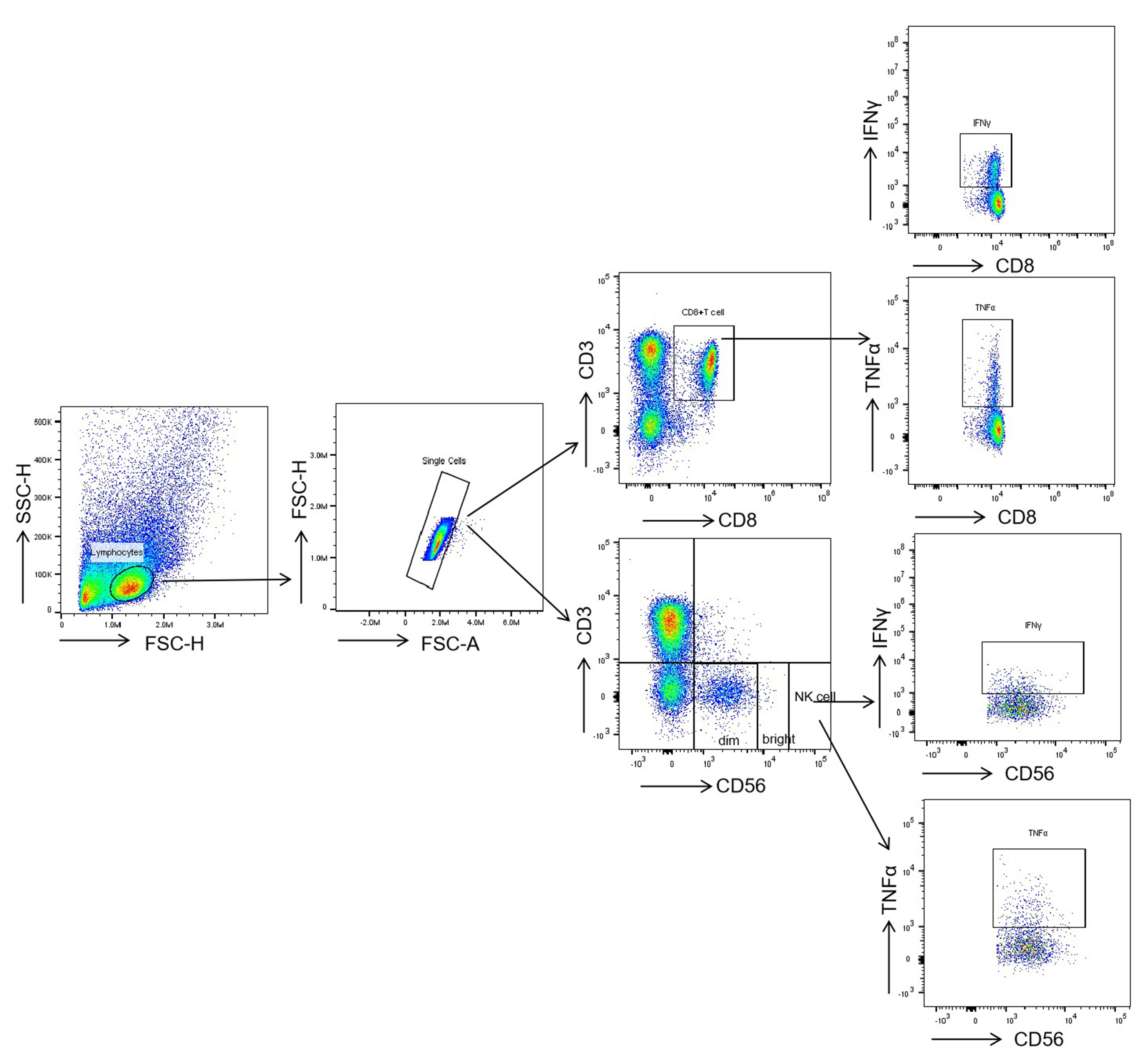

Supplement: Supplementary Figure S4 — Representative flow plots of the gating strategy used for functionality assessment of NK cells and CD8+ T cells. [file Image_4.JPEG]
