# Supplementary material for: The Characteristics of Natural Killer Cells and T Cells Vary With the Natural History of Chronic Hepatitis B in Children
Source: Front Pediatr. 2021 Nov 25;9:736023. doi: 10.3389/fped.2021.736023 (PMC8656424; doi:10.3389/fped.2021.736023)
Supplement: Supplementary Table S1 — Univariate and multivariate analysis of variables associated with clinical phases (IA phase vs. IT phase) in CHB children. [file Table_1.docx]

**Table S1. Univariate and multivariate analysis of variables associated with clinical phases(IA phase vs IT phase) in CHB children**

| Variables | Univariate analysis | | *P* | Multivariate analysis | | *P* |
| --- | --- | --- | --- | --- | --- | --- |
|  | OR(95%CI) | B |  | OR(95%CI) | B |  |
| %CD38+/CD8+T | 1.05(1.01,1.09) | 0.05 | **0.008** | 1.12(1.01,1.25) | 0.11 | **0.039** |
| %CD38+/CD4+T | 0.98(0.95,1.01) | -0.02 | 0.232 | 0.86(0.75,0.99) | -0.15 | **0.033** |
| %HLA-DR+/NK | 1.04(1.01,1.07) | 0.04 | **0.011** | 1.01(0.94,1.08) | 0.01 | 0.791 |
| %NKp46+/NK | 1.01(0.99,1.03) | 0.01 | 0.418 | 1.00(0.93,1.07) | 0.00 | 0.925 |
| %NKp30+/NK | 1.01(0.99,1.03) | 0.01 | 0.468 | 1.02(0.97,1.08) | 0.02 | 0.476 |
| gender（female vs male#) | 1.53(0.69,3.38) | 0.42 | 0.296 | 1.49(0.33,6.84) | 0.40 | 0.608 |
| age | 1.03(0.93,1.14) | 0.03 | 0.583 | 0.86(0.61,1.19) | -0.16 | 0.354 |
| Genotype(C vs B#) | 1.01(0.32,3.20) | 0.01 | 0.984 | 1.23(0.27,5.61) | 0.21 | 0.788 |

# represents the reference group; OR=odds ratio.
