# Supplementary material for: The Characteristics of Natural Killer Cells and T Cells Vary With the Natural History of Chronic Hepatitis B in Children
Source: Front Pediatr. 2021 Nov 25;9:736023. doi: 10.3389/fped.2021.736023 (PMC8656424; doi:10.3389/fped.2021.736023)
Supplement: Supplementary Table S2 — Comparison of immune characteristics between gray zone and strict IT phase. [file Table_2.docx]

**Table S2. Comparison of immune characteristics between gray zone and strict IT phase**

| **Frequency(%)**  median(P25, P75) | **strict IT phase**  **(<1ULN ALT)** | **Gray zone of IT phase**  **(1-1.5ULN ALT)** | ***P*** | ***Z*** |
| --- | --- | --- | --- | --- |
| CD56^bright^+/NK cells | 3.47(1.99,5.23) | 6.58(3.12,9.64) | **0.003** | 2.95 |
| CD56^dim^ +/NK cells | 96.55(94.78,98.00) | 93.45(90.35,96.85) | **0.003** | -3.00 |
| NKp30+/NK cells | 49.10(32.68,66.85) | 64.30(52.33,71.75) | 0.081 | 1.74 |
| NKp46+/NK cells | 56.90(37.25,65.45) | 54.05(35.55,74.35) | 0.553 | 0.59 |
| HLA-DR+/NK cells | 26.10(12.63,34.28) | 21.05(15.5,26.18) | 0.559 | -0.58 |
| CD4+/T cells | 44.50(40.30,53.90) | 48.80(46.68,57.25) | **0.027** | 2.22 |
| CD8+/T cells | 39.00(31.70,45.30) | 38.60(30.33,45.35) | 0.750 | -0.32 |
| CD38+/CD4+T cells | 74.25(60.80,81.70) | 80.95(73.65,87.75) | 0.060 | 1.88 |
| CD38+/CD8+T cells | 66.15(54.73,76.38) | 74.30(65.45,86.33) | **0.041** | 2.04 |

IT: immune tolerant; ALT: alanine aminotransferase.
