# Supplementary material for: The Characteristics of Natural Killer Cells and T Cells Vary With the Natural History of Chronic Hepatitis B in Children
Source: Front Pediatr. 2021 Nov 25;9:736023. doi: 10.3389/fped.2021.736023 (PMC8656424; doi:10.3389/fped.2021.736023)
Supplement: Supplementary Table S3 — Correlation analysis of the activating NK and T cell subsets with serum HBV DNA and ALT levels. [file Table_3.docx]

| Phase | Items | ALT/ULN | | HBV DNA(log_10_IU/ml) | |
| --- | --- | --- | --- | --- | --- |
|  |  | r(95%CI) | *P* | r(95%CI) | *P* |
| IT | Bright/dim ratio | 0.40(0.15,0.61) | **0.002** | -0.07(-0.34,0.20) | 0.601 |
|  | %NKp30+/NK cells | 0.19(-0.08,0.44) | 0.155 | 0.14(-0.14,0.40) | 0.300 |
|  | %NKp46+/ NK cells | 0.11(-0.16,0.37) | 0.409 | 0.18(-0.09,0.43) | 0.181 |
|  | %HLA-DR+/NK cells | 0.02(-0.25,0.29) | 0.883 | -0.06(-0.33,0.22) | 0.665 |
|  | %CD38+/CD4+T cells | 0.15(-0.14,0.42) | 0.287 | 0.30(0.01,0.54) | **0.036** |
|  | %CD38+/CD8+T cells | 0.30(0.01,0.54) | **0.037** | 0.28(-0.01,0.52) | **0.049** |
| IA | Bright/dim ratio | -0.01(-0.31,0.30) | 0.972 | -0.11(-0.40,0.20) | 0.489 |
|  | %NKp30+/NK cells | -0.12(-0.41,0.19) | 0.432 | 0.17(-0.14,0.45) | 0.262 |
|  | %NKp46+/ NK cells | -0.07(-0.37,0.24) | 0.640 | 0.03(-0.28,0.33) | 0.868 |
|  | %HLA-DR+/NK cells | 0.46(0.18,0.67) | **0.002** | -0.29(-0.55,0.01) | **0.049** |
|  | %CD38+/CD4+T cells | 0.13(-0.19,0.43) | 0.395 | 0.16(-0.16,0.45) | 0.312 |
|  | %CD38+/CD8+T cells | 0.223(-0.10,0.50) | 0.155 | -0.27(-0.53,0.05) | 0.088 |

**Table S3. Correlation analysis of the activating NK and T cell subsets with serum HBV DNA and ALT levels**

IA: immune active; IT: immune tolerant; ALT: alanine aminotransferase; CI:confidence interval.
